# COVID-19 ORF3a Viroporin-Influenced Common and Unique Cellular Signaling Cascades in Lung, Heart, and the Brain Choroid Plexus Organoids with Additional Enriched MicroRNA Network Analyses for Lung and the Brain Tissues

**DOI:** 10.1021/acsomega.3c06485

**Published:** 2023-11-17

**Authors:** Soura Chakraborty, Shrabonti Chatterjee, Subhashree Mardi, Joydeep Mahata, Suneel Kateriya, Pradeep Punnakkal, Gireesh Anirudhan

**Affiliations:** † School of Biotechnology, Jawaharlal Nehru University, New Mehrauli Road, New Delhi 110067, India; ‡ Integrated Science Education and Research Centre (ISERC), Institute of Science (Siksha Bhavana), Visva Bharati (A Central University), Santiniketan (P.O.), Birbhum (DT), West Bengal 731235, India; § Department of Biophysics, Postgraduate Institute of Medical Education & Research (PGIMER), Chandigarh 160012, India

## Abstract

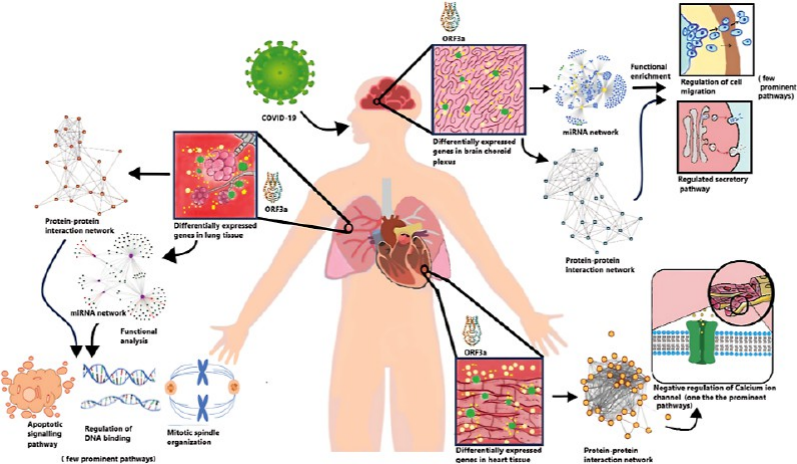

Tissue-specific implications of SARS-CoV-2-encoded accessory proteins are not fully understood. SARS-CoV-2 infection can severely affect three major organs—the heart, lungs, and brain. We analyzed SARS-CoV-2 ORF3a interacting host proteins in these three major organs. Furthermore, we identified common and unique interacting host proteins and their targeting miRNAs (lung and brain) and delineated associated biological processes by reanalyzing RNA-seq data from the brain (COVID-19-infected/uninfected choroid plexus organoid study), lung tissue from COVID-19 patients/healthy subjects, and cardiomyocyte cells-based transcriptomics analyses. Our in silico studies showed ORF3a interacting proteins could vary depending upon tissues. The number of unique ORF3a interacting proteins in the brain, lungs, and heart were 10, 7, and 1, respectively. Though common pathways influenced by SARS-CoV-2 infection were more, unique 21 brain and 7 heart pathways were found. One unique pathway for the heart was negative regulation of calcium ion transport. Reported observations of COVID-19 patients with a history of hypertension taking calcium channel blockers (CCBs) or dihydropyridine CCBs had an elevated rate of intubation or increased rate of intubation/death, respectively. Also, the likelihood of hospitalization of chronic CCB users with COVID-19 was greater in comparison to long-term angiotensin-converting enzyme inhibitors/angiotensin receptor blockers users. Further studies are necessary to confirm this. miRNA analysis of ORF3a interacting proteins in the brain and lungs revealed 3 of 37 brain miRNAs and 1 of 25 lung miRNAs with high degree and betweenness indicating their significance as hubs in the interaction network. Our study could help in identifying potential tissue-specific COVID-19 drug/drug repurposing targets.

## 1 Introduction

The risk of SARS-CoV-2 (COVID-19) that caused the recent pandemic is far from being over, because of the lack of complete protection by the current vaccines and ever-emerging variants. This situation is more alarming due to the detection of COVID-19 antibodies in a wide variety of animals both domestic and wild as well as the detection of COVID-19 infection itself, generating a possibility of these animals becoming potential as well as observed reservoir hosts.^1−5^ In addition, observation of unique COVID-19 clades specific to mink as well as variations observed among different strains^6^ suggests the emergence of new variants independently in these hosts that could pose an increased risk to humans. Also, the lack of effective treatment targeting COVID-19 is a major lacuna that impairs our combat against it, including the development of long COVID. COVID-19, with a positive-strand RNA as its genetic material, is a member of the Coronaviridae family that causes many diseases in vertebrates including humans. In 2002, severe acute respiratory syndrome (SARS) caused by SARS-CoV was identified in humans with a mortality rate of 15% all over the world^7^ and Middle East respiratory syndrome (MERS) caused by MERS-CoV in 2012 with a mortality rate of 35%^8^ are coronaviruses.

Initially, SARS-CoV-2 pathogenesis was focused on respiratory pathologies, mainly on the symptoms including cough, fever, common cold, and respiratory distress. However, recent evidence has shown that SARS-CoV-2 is not only confined to the organs of the respiratory system but also invades other organs such as the brain, heart, kidney, intestine, etc.^9−13^ For the development of an effective treatment against COVID-19, investigating the mechanism of action of different virally encoded proteins is paramount. This is envisaged in the use of spike protein as a vaccine candidate, as well as testing putative drug molecules targeting RNA-dependent RNA polymerase, viral proteases 3CLpro and PLpro, etc.^14−19^

Viroporins are small channel-forming integral membrane viral proteins present in many different viruses (reviewed in Scott and Griffin).^20^ They vary in their number of amino acids, transmembrane domains (1–3), ion selectivity (H^+^, Na^+^, K^+^, Ca^2+^, and Cl^–^), and have diverse functions depending upon the virus family they belong to (reviewed in Scott and Griffin).^20^ Diverse functional roles of viroporins in different viral families are observed including aiding in cell entry, cell lysis, particle production, viral spread, as the TNF antagonist, influencing and manifesting pathogenesis, and mitochondrial permeability (reviewed in Scott and Griffin).^20^ Adamantane inhibitors, used in the treatment of influenza A virus, targeting M2 protein^21−25^ are halted because of resistant polymorphisms present in the majority of circulating strains (reviewed in Scott and Griffin).^20^ Another viroporin targeted was the p7 protein of the hepatitis C virus, where the inhibitors fall into three categories, viz., adamantanes, alkyl imino-sugars, and hexamethylene amiloride. Recombinant proteins or peptides were used in vitro for their identification.^20,26−28^ Though genotype-dependent resistance was observed,^29,30^ broad-spectrum ligands targeting multiple targets could be explored as an option to overcome this resistance.^31^ Warranting more studies, inhibition of dengue virus replication by amantadine was reported by an in vitro study^32^ and administration of amantadine at the onset and 2 to 6 days after onset was observed to diminish the symptoms of dengue infection in comparison to the control group.^33^ Hence looking for viroporin-interacting proteins and different pathways would be an alternative strategy for therapeutic intervention.

Viroporins associated with coronaviruses are E, 3a, ORF8a, and ORF4a.^20,34^ In SARS-CoV-2, ORF3a is one of the putative viroporins. When purified from heterologous expression systems, it is reported to exist as a 62 kDa dimer and 124 kDa tetramer with each protomer having 3 transmembrane domains.^35^ ORF3a induces inflammatory response in the host^36−39^ and also mediates optimal replication.^40^ Retrospective analyses of 16 studies for the levels of inflammatory markers like C-reactive protein (CRP), procalcitonin (PCT), serum ferritin, erythrocyte sedimentation rate (ESR), and interleukin-6 (IL-6) showed positive correlation with COVID-19 severity.^41^ Antibody response is observed against SARS-CoV ORF3a^42,43^ and SARS-CoV-2 ORF3a.^44,45^ Recently, the role of viroporin ORF3a in the induction of NLRP3-mediated inflammatory responses has also been reported.^36,39^ It is also observed that SARS-CoV-2 ORF3a viroporin has a relatively weaker proapoptotic activity compared to the ORF3a viroporin of SARS-CoV when expressed in cell lines.^46^ It is also speculated that relatively mild infection of the SARS-CoV-2 might give it an advantage in spreading.^46^ Observation of SARS-CoV-2 ORF3a unlike SARS-CoV ORF3a promoting lysosomal exocytosis-mediated viral egress,^47^ blocking autolysosome formation by interfering with the assembly of STX17-SNAP29-VAMP8 SNARE complex,^48,49^ and mutation in ORF3a associated with increased mortality rate in SARS-CoV-2 infection increase its importance.^50^

This warrants the investigation of ORF3a and(or) other interacting partners involved in various pathways as a plausible drug target(s). The targeted disruption at the interface of the interaction of viroporin and the host protein might also be a useful strategy to overcome drug resistance. In our in silico analyses reported here, we attempted to find out various pathways where SARS-CoV-2 ORF3a and its interacting partners are involved. Common and unique pathways in the lung, heart, and brain choroid plexus organoids were found in addition to the observation that SARS-CoV-2 ORF3a interacting partners get regulated after SARS-CoV-2 infection. We were able to find 10, 7, and, 1 unique interacting proteins out of 200, 197, and 175 interacting proteins that were regulated in the brain choroid plexus organoids, lung, and heart, respectively, after SARS-CoV-2 infection. Looking for probable biological processes of the brain and lung regulated by miRNAs, we analyzed miRNet for interacting miRNAs of 8 proteins in these tissues that interact with ORF3a. We could find 2 out of 37 miRNAs in the brain and 1 out of 25 miRNAs in the lungs with high degree and betweenness that signifies the role of these miRNAs as hubs. We also looked for SARS-CoV-2-influenced miRNAs as well as proteins that interact with ORF3a interacting proteins in a tissue-specific manner in the brain and lung to find the prominent biological processes in these tissues.

## 2 Materials and Methods

### 2.1 SARS-CoV-2 ORF3a-Interacting Human Proteins

To find out the influence of SARS-CoV-2 ORF3a in human cells, we sought to obtain cellular proteins interacting with SARS-CoV-2 ORF3a viroporin from the Human Protein Atlas and collected information about SARS-CoV-2 interacting human proteins.^51^ SARS-CoV-2 ORF3a viroporin interacts with eight human cellular proteins. To find out the tissue-specific effect of ORF3a viroporin, we started our analysis with these eight proteins, identified tissue-specific proteins and miRNAs interacting with these eight proteins, and analyzed Gene Ontology (GO) term enrichment analysis to identify biological processes associated with these interacting networks.

### 2.2 Identification of Hub Genes and Clustering of ORF3a Interacting Proteins Network

ORF3a interacting genes that are common in the brain, heart, and lung were used to construct protein–protein interaction in STRING version 11.0.^52^ This network was further grown to obtain more interactions. The whole network was uploaded in Cytoscape version 3.9.0,^53^ and hub genes for different networks were found using the cytoHubba plugin.^54^ In our study, node scores of hub genes were calculated on the basis of the Maximum Clique Centrality (MCC), bottleneck, and EcCentricity algorithm separately. Protein–protein interaction networks of tissue-specific common proteins in the heart, lung, and brain were further clustered into groups to find highly connected regions in the network using the MCODE^55^ application in Cytoscape. K-core cutoff 2 was used with a maximum depth of 100. K-core denotes the score deviance from the seed node’s score for expanding the cluster. Maximum depth is the limit by which the search distance from the seed is set. The Cluster module network was separately analyzed to find the biological processes regulated by seed proteins in clusters. BiNGO (Biological Networks Gene Ontology)^56^ tool in Cytoscape was used for assessing statistically over-represented biological processes regulated by these clusters with a significance value of 0.05 as the cutoff. The hypergeometric test was used as a statistical test, and Benjamini and Hochberg’s false discovery rate (FDR) correction was used for multiple testing correction.

### 2.3 Tissue-Specific Protein Interaction Network Construction

Tissue-specific interacting protein partners of these eight proteins have been isolated from IID (Integrated Interactions Database) (http://ophid.utoronto.ca/iid) based on two or more experimental pieces of evidence (studies or bioassays).^57^ The experiments in which these interactions were established are provided in the Supporting Information along with PubMed IDs. Tissue-specific common and unique proteins (provided in the Supporting Information) have been identified through Venn diagram analysis using http://genevenn.sourceforge.net/. Tissue-specific interacting proteins were used for further analysis. GO enrichment analysis was performed using the R package clusterProfiler.^58^ The Benjamini and Hochberg test had been used for pAdjustMethod (for the FDR correction). Enrichments with *p*-value ≤0.01 had been considered as significant. Enrichment for the common pathways with *q*-value ≤0.01 was considered as significant.

### 2.4 Tissue-Specific miRNA Network Construction

ORF3a may regulate the activities of a diverse array of miRNAs. miRNAs are well-known influencers of gene expression. Using miRNet (https://www.mirnet.ca),^59^ we have identified and constructed a tissue-specific interaction network of ORF3a interacting 8 proteins with miRNAs. Based on the availability of data in miRNet, we only constructed miRNA networks for the brain and lung. The degree of a node has been calculated by the number of other nodes it connected, and betweenness centrality signifies the bridging role of a node in a network. We have used the GO database for functional enrichment analysis of the miRNA network and identified significant (*p* < 0.05) biological processes associated with the miRNA networks. Enrichment analysis was based on the hypergeometric tests after adjustment for FDR.

Likewise, as SARS-CoV-2 influences miRNAs, we wanted to study the miRNA–mRNA network involving these miRNAs and SARS-CoV-2-influenced proteins interacting with SARS-CoV-2 ORF3a binding proteins. Common miRNAs in the reported list of SARS-CoV2-influenced circulating miRNAs^60^ and the miRNAs that can target brain-expressing interacting partners of SARS-CoV-2 ORF3a interacting partners were taken for analysis. Proteins taken for analysis were SARS-CoV-2-influenced SARS-CoV-2 ORF3a interacting proteins expressed in either the brain or lung.

### 2.5 Delineating Tissue-Specific Effect of ORF3a from RNA-seq Data

Based on our above analysis, we sought to reanalyze published RNA-seq data to further delineate the tissue-specific effect of ORF3a. So, we chose three different data sets for the brain (choroid plexus organoids study),^61^ lung (COVID-19 infected lung tissue compared to healthy subject),^62^ and heart-cardiomyocytes based SARS-CoV-2 transcriptomics study.^63^ Differentially expressed genes with *p*-value ≤0.05 had been considered for further analysis. From these data sets, we selectively identified significant differentially expressed genes encoding proteins that directly or indirectly interact with SARS-CoV-2 ORF3a (here we considered the protein list depicted in Table 1 as SARS-CoV-2 ORF3a interacting protein partners). Then we performed GO enrichment analyses to find relevant biological pathways in a similar way as depicted earlier. Enrichments with *p*-value ≤0.01 had been considered as significant.

**Table 1:** ORF3a Directly Interacting Proteins

	user ID	ensemble gene ID	symbol	gene type	species	Chr	position (Mbp)
1	CLCC1	ENSG00000121940	CLCC1	protein_coding	human	1	108.9274
2	ARL6IP6	ENSG00000177917	ARL6IP6	protein_coding	human	2	152.7179
3	TRIM59	ENSG00000213186	TRIM59	protein_coding	human	3	160.4324
4	VPS11	ENSG00000160695	VPS11	protein_coding	human	11	119.0678
5	ALG5	ENSG00000120697	ALG5	protein_coding	human	13	36.94974
6	VPS39	ENSG00000166887	VPS39	protein_coding	human	15	42.1587
7	HMOX1	ENSG00000100292	HMOX1	protein_coding	human	22	35.38036
8	SUN2	ENSG00000100242	SUN2	protein_coding	human	22	38.73473

**Figure 1:**
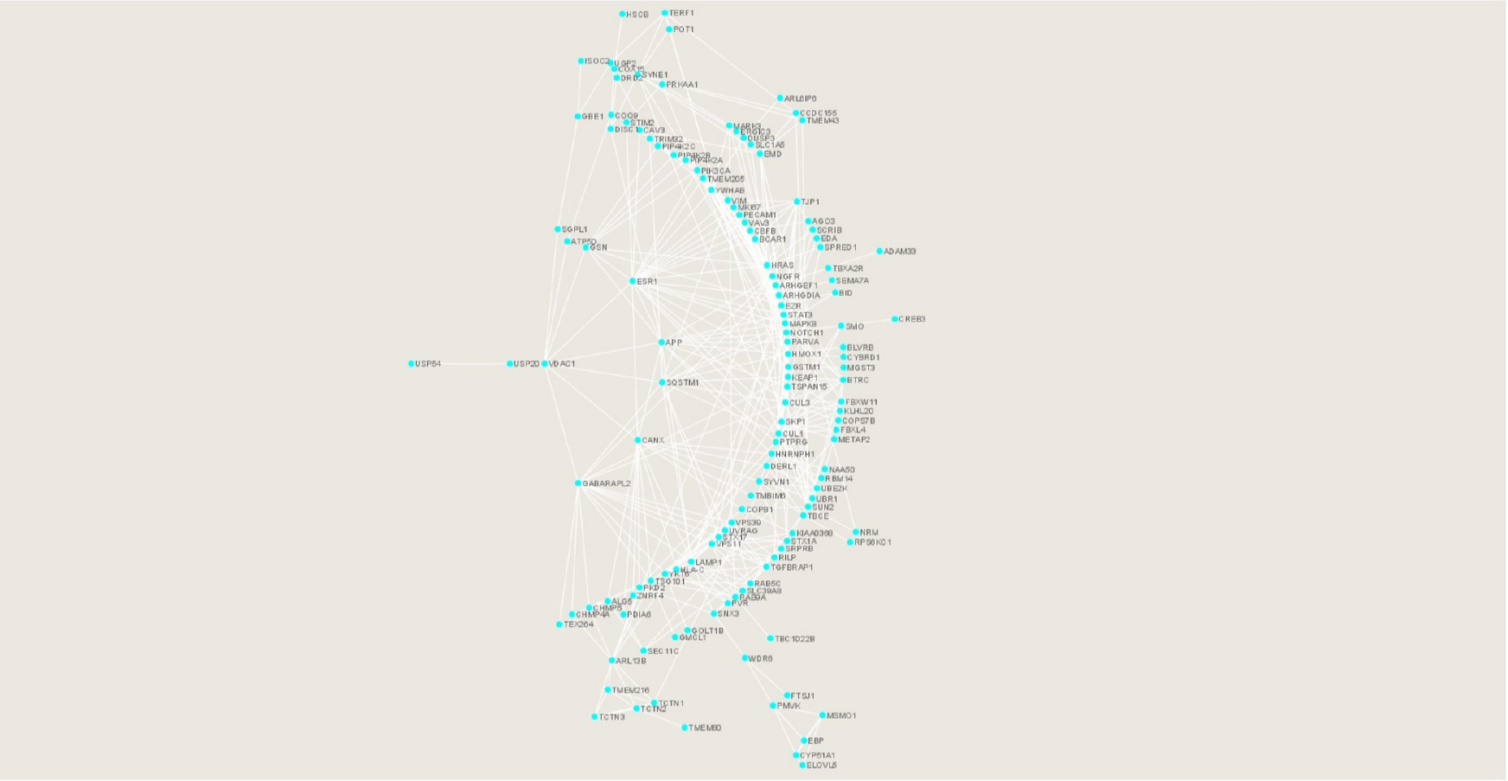
Network presentation of regulated common proteins expressed in the brain, lungs, and heart after SARS-CoV-2 infection. Visualization done in Cytoscape.

**Figure 2:**
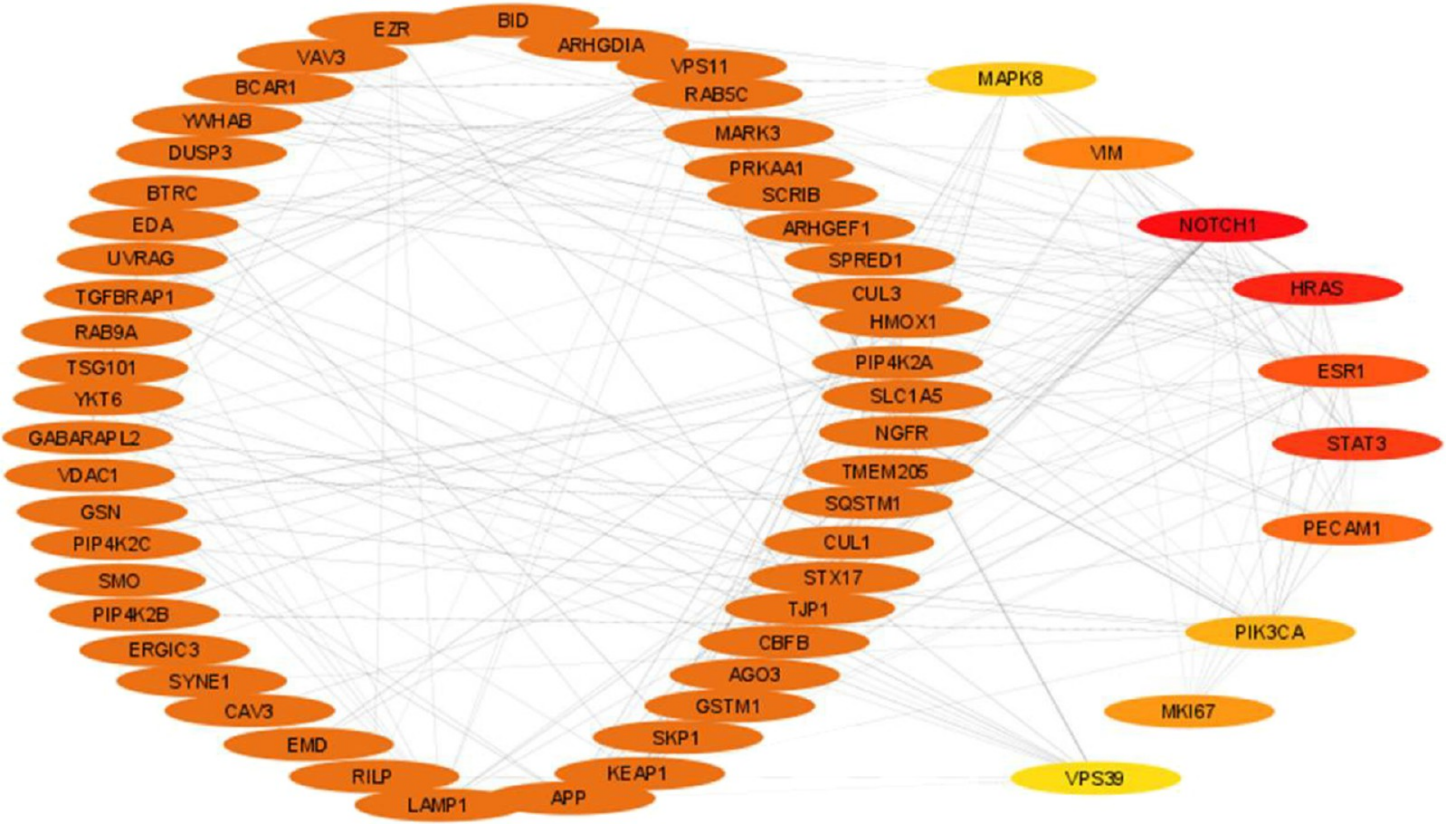
Top 10 hub genes of the common protein networks of regulated proteins after SARS-CoV-2 infection in the brain, lungs, and heart. Regulated proteins common in the brain, lungs, and heart were analyzed by cytoHubba to find the top 10 hub genes (shown separately from the circularly arranged proteins). Node color from red to yellow represents higher to lower significance. NOTCH1, HRAS, STAT3, and ESR1 are highly significant major hub genes.

## 3 Results

### 3.1 ORF3a Interacting Protein Diversity Varies in Different Tissues

SARS-CoV-2 affects various organs in different ways. To know its effect in the brain, lungs, and heart, protein–protein interaction studies were performed with proteins interacting with eight ORF3a proteins. Proteins that were influenced by SARS-CoV-2 and those proteins that were expressed in the brain, lung, and heart were included in the analyses. These analyses from the experiment-based tissue-specific interaction network revealed 199, 197, and 175 interacting proteins in the brain choroid plexus organoids, lung, and heart, respectively. Venn diagram analysis revealed that 163 of these interacting proteins were common in all three tissues. The analysis revealed ten unique interacting proteins (BLZF1, CDK5, ELOVL4, LRSAM1, NECAB2, SCARA3, SEC22A, TMEM17, UGT8, and ZDHHC22) in the brain, seven unique interacting proteins (CHAT, CLDN4, CRB3, EVC2, PCDHB7, PDZK1IP1, and UNC93B1) in the lung, and only one unique interacting protein (EPN3) in the heart (Supporting Information Data 1).

### 3.2 Analysis of SARS-CoV-2-Influenced ORF3a Interacting Common Proteins in the Brain, Lung, and Heart Predicted Hub Genes and Clusters in the Protein Network

To find the prominent players in the brain, lung, and heart, we subjected the common SARS-CoV-2-influenced proteins to protein network identification. The protein–protein networks constructed consisted of 168 nodes and 387 edges, with an average node degree of 4.61. Standard *p*-value ≤0.05 was used as cut off (Figure 1). The top 10 hub genes in the common proteins network identified by the MCC algorithm were NOTCH1, HRAS, STAT3, ESR1, PECAM1, VIM, MKI67, PIK3CA, MAPK8, and VPS38 (Figure 2), whereas according to the bottleneck algorithm, the top 10 hub genes identified were NOTCH1, HRAS, PIK3CA, LAMP1, ESR1, MAPK8, KEAP1, GSN, ARHGDIA, and YWHAB (Supporting Information Data 2). NOTCH1 and HRAS showed maximum significance among all hub genes. EcCenrtricity algorithm was also employed, and the top 10 hub genes identified were VDAC1, ESR1, MAPK8, BID, EZR, KEAP1, APP, BCAR1, ARHGEF1, and SQSTM1 that had the same rank (1.0) and score (0.333) (Supporting Information Data 2).

**Figure 3:**
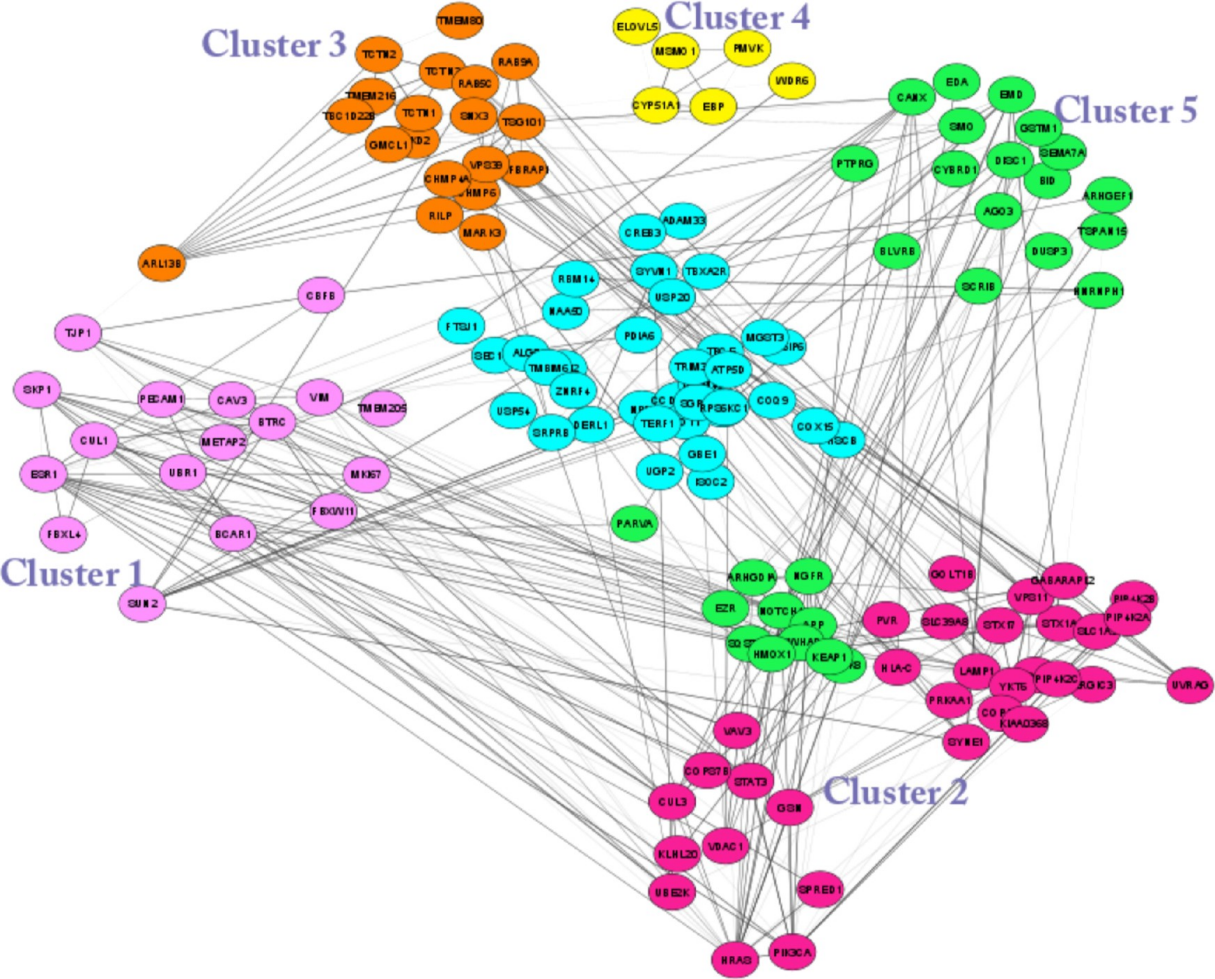
MCODE analysis of protein–protein interactome representing the top five clusters. All five clusters are highly connected and each cluster is represented by a different color. Individual clusters are depicted in Figures S1 to S5.

With the same data that we used to find the hub genes, MCODE was utilized to interpret highly connected top 5 clusters from the network (Figure 3 and Supporting Information Data 3). To elicit the biological processes associated with every cluster, we used BiNGO. Cluster 1 had a maximum score of 7.707 with two seed proteins, proliferation marker protein *K*_i_-67 (MKI67) and Ezrin (EZR) (Figure S1). Biological processes associated with them are the regulation of cell death and apoptosis. Cluster 2 had a maximum score of 6.182 with two seed proteins—UV radiation resistance-associated gene (UVRAG) and EZR (Figure S2). Maximum proteins in this complex regulate protein localization, transport, and intracellular signal transduction. Both Cluster 3 (Figure S3) and Cluster 4 (Figure S4) had a score value of 3.600, whereas for Cluster 5 (Figure S5) it was 3.481. Seed proteins for them were tectonic family member 2 (TCTN2) (Cluster 3), methylsterol monooxygenase 1 (MSMO1) (Cluster 4), and again EZR for Cluster 5. Biological processes associated with Cluster 3 are protein transport, establishment of protein localization, and other macromolecule localization. Cluster 4 is associated with regulation of cholesterol and sterol, the steroid biosynthesis process, and lipid and alcohol biosynthesis. Biological processes related to all five clusters are given in Supporting Information Data 4.

**Figure 4:**
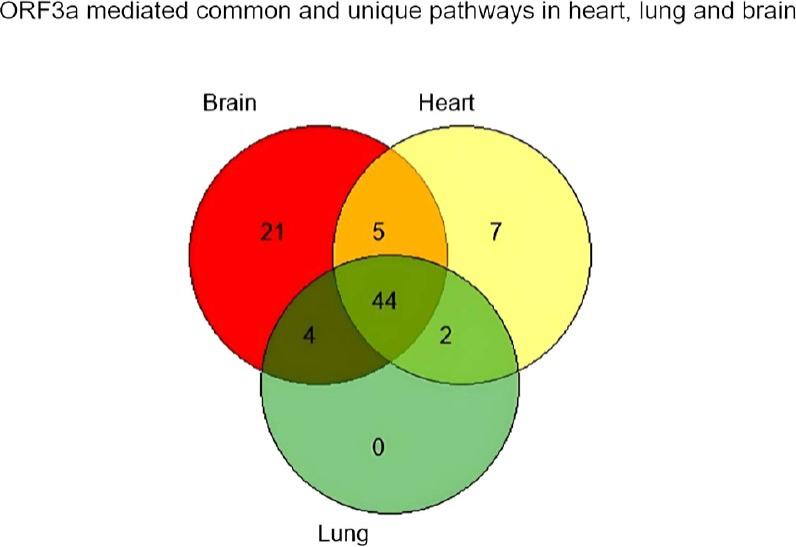
Venn diagram showing the number of biological pathways associated with ORF3a interacting proteins in the heart, lung, and brain. 44 pathways are common in the heart, lungs, and brain. 21 unique pathways were observed in the brain, whereas in the heart, 7 were present. No unique pathway was observed in the lungs.

**Figure 5:**
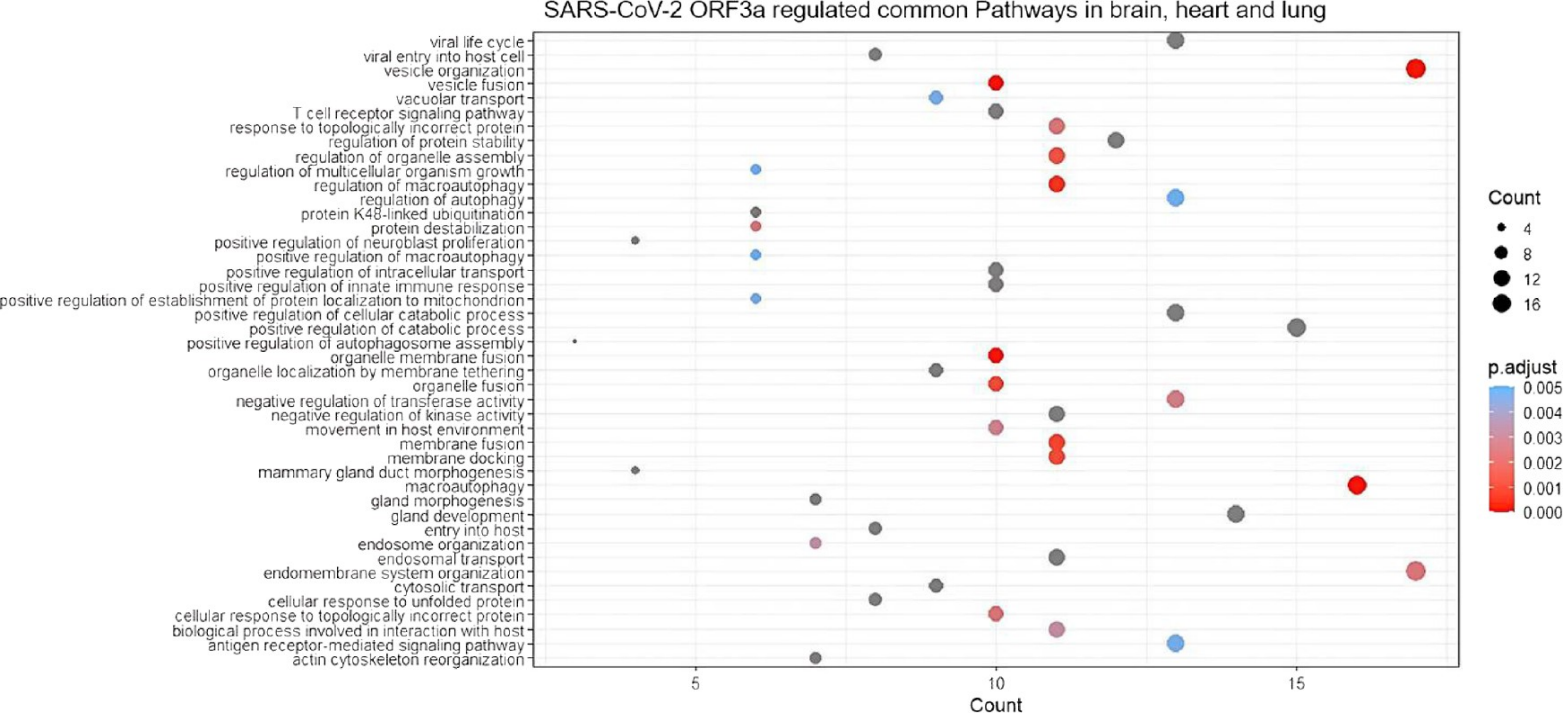
SARS-CoV-2 ORF3a regulated common pathways in the lung, heart, and brain. The dot size represents the gene ratio and the dot color depicts the *p*.adjust value as in the heat map.

### 3.3 ORF3a Interacting Proteins Can Influence General and Unique Tissue-Specific Biological Processes

**Figure 6:**
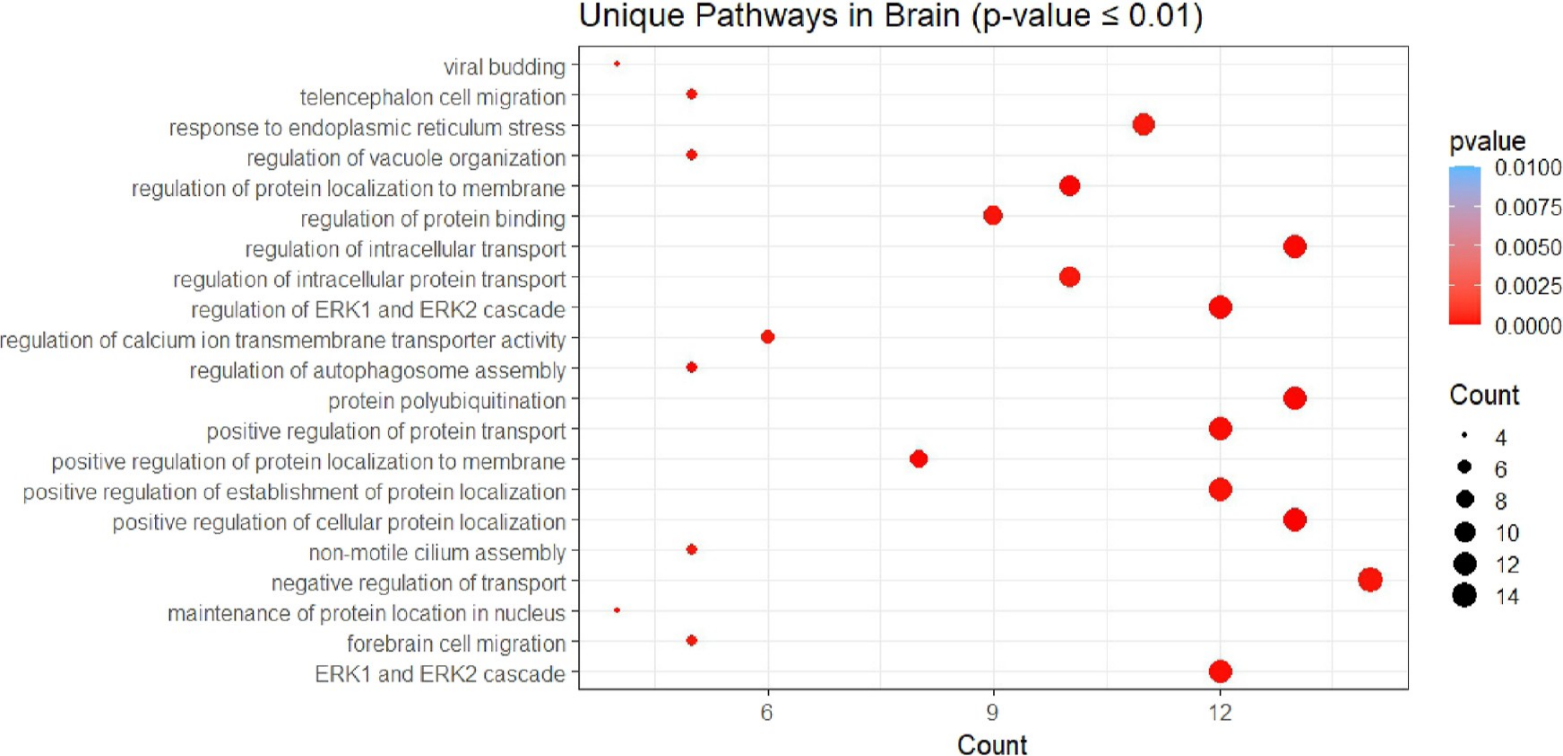
SARS-CoV-2 ORF3a regulates unique pathways in the brain. Dot size represents the gene ratio and dot color depicts the *p*-value as in the heat map.

**Figure 7:**
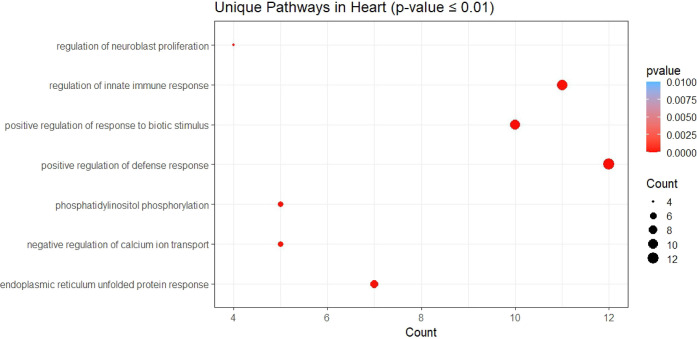
SARS-CoV-2 ORF3a regulates unique pathways in the heart. Dot size represents the gene ratio and dot color depicts the *p*-value as in the heat map.

In general, ORF3a viroporin influences vesicular transport, cytoskeleton organization, protein kinase signaling, protein stability regulation, and ubiquitination. Tissue-specific interacting protein partners were used to elucidate the related pathways that are probable and susceptible to COVID-19 infections in the heart, brain, and lung. GO enrichment analyses revealed no unique pathway in the lung when compared with the brain and heart. On the other hand, among these three major organs (heart, lung, and brain), ORF3a-mediated unique pathways were observed to be more in the brain than in the heart (Figure 4). Common pathways influenced by ORF3a in all three organs are shown in Figure 5. In the brain, ORF3a-influenced unique pathways include telencephalon and forebrain cell migration, intracellular transport, ERK1 and ERK2 cascade, controlling protein ubiquitination and localization, and nonmotile cilium assembly. Viral budding was one of the pathways observed to be affected only in the brain (Figure 6) (Supporting Information Data 5 and 6). In the heart, ORF3a can also influence some unique biological pathways including the regulation of phosphatidyl inositol pathway, unfolded protein response in ER, and can positively regulate biotic stimulus and defense response. Among these, the negative regulation of calcium ion transport by ORF3a interacting proteins can adversely affect cardiac function (Figure 7).

**Figure 8:**
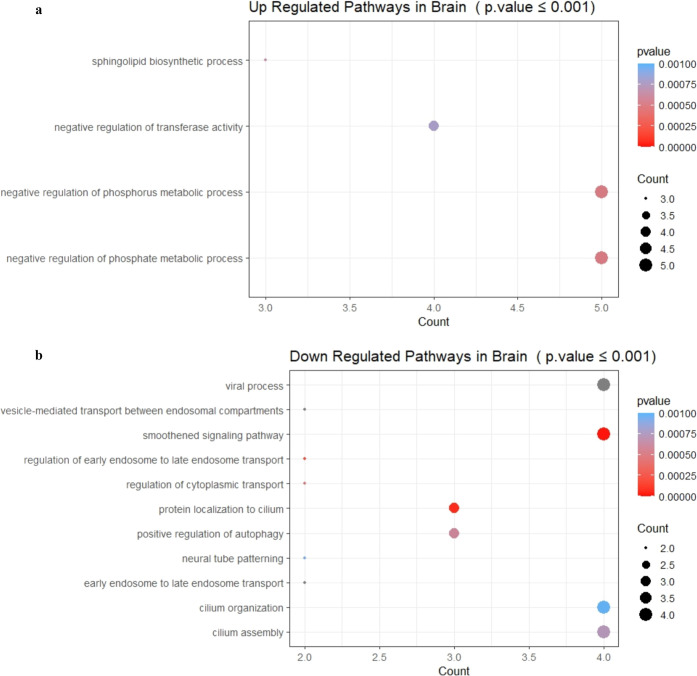
Dot plot of enriched GO terms of differentially expressed genes in the brain. The *Y*-axis indicates the GO term and the *X*-axis shows the count of genes per GO term. The color gradient indicates the *p*-value, using the Benjamini–Hochberg method. (a) Upregulated genes and (b) downregulated genes.

**Figure 9:**
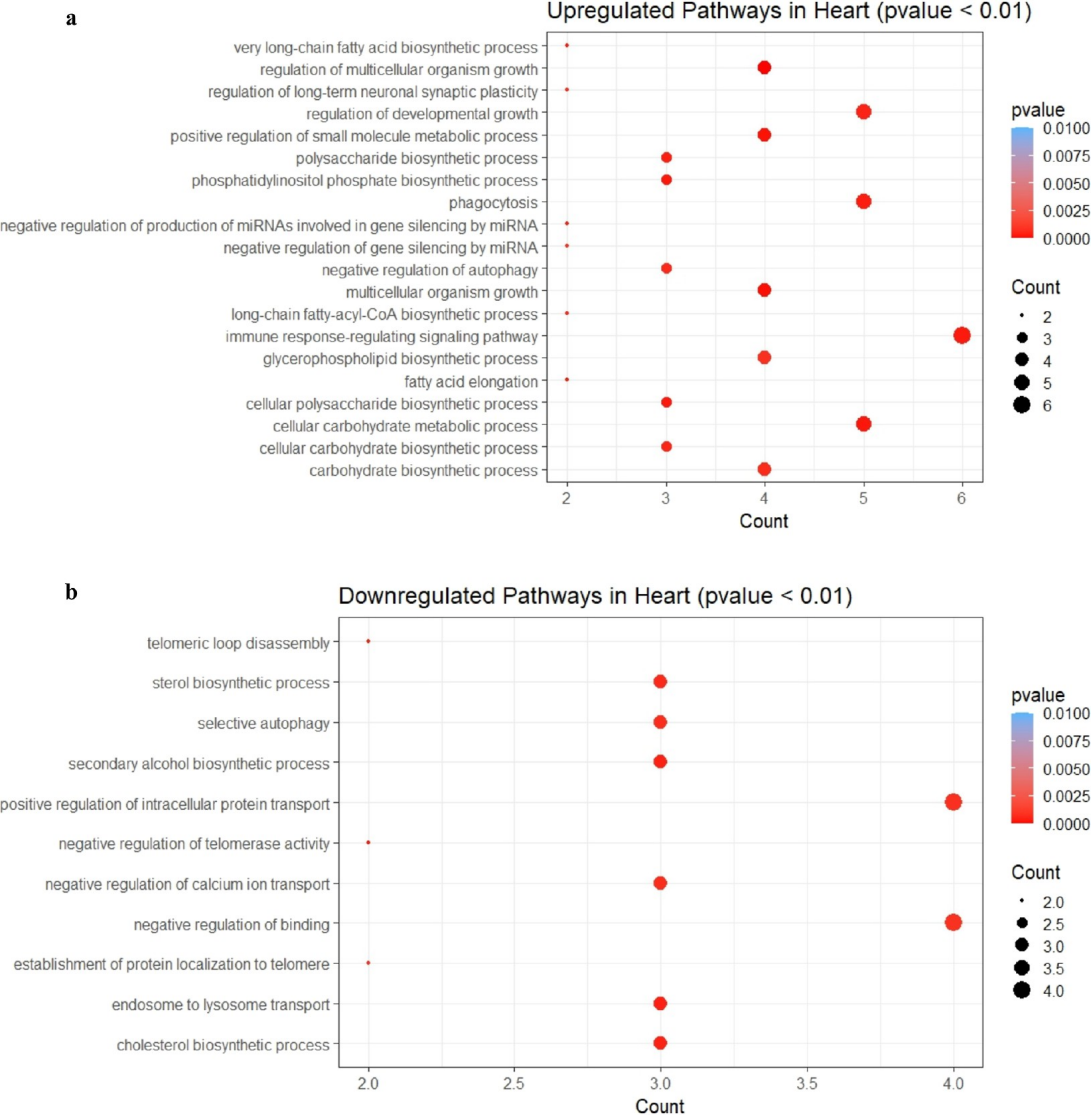
Dot plot of enriched GO terms of differentially expressed genes in the heart. The *Y*-axis indicates the GO term and the *X*-axis shows the count of genes per GO term. The color gradient indicates the *p*-value, using the Benjamini–Hochberg method. (a) Upregulated genes and (b) downregulated genes.

**Figure 10:**
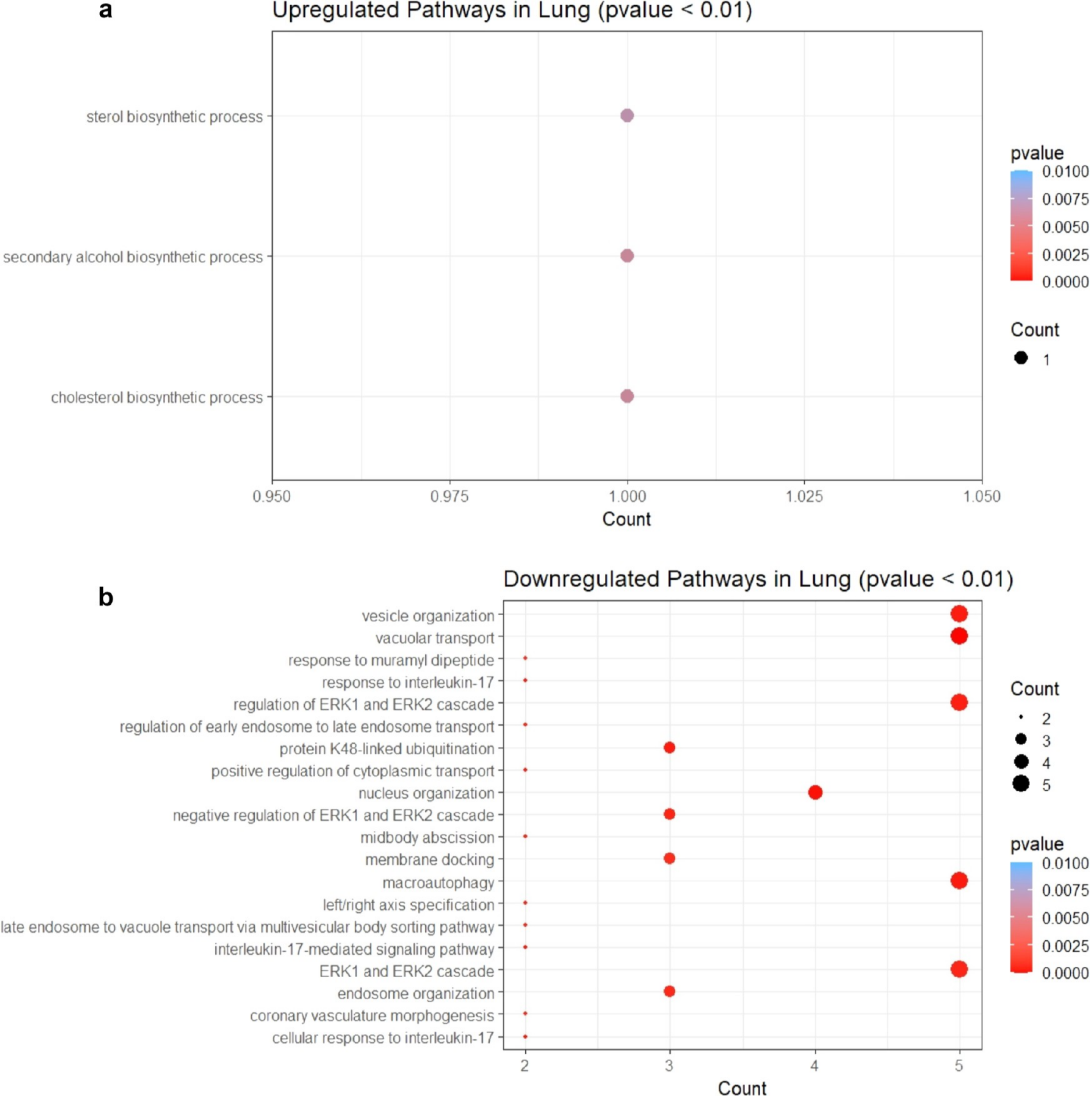
Dot plot of enriched GO terms of differentially expressed genes in the lung. The *Y*-axis indicates the GO term and the *X*-axis shows the count of genes per GO term. The color gradient indicates the *p*-value, using the Benjamini–Hochberg method. (a) Upregulated genes and (b) downregulated genes.

Next, we wanted to analyze both upregulated and downregulated pathways based on SARS-CoV-2-influenced differentially expressed genes in a tissue-specific manner. We delineated up- and downregulated genes to find out the pathways they influence. In the brain choroid plexus organoid study, among upregulated genes-influenced pathways, prominent ones were negative regulation of phosphorylation, negative regulation of phosphate and phosphorus metabolic processes, and cellular response to peptide (Figure 8a), whereas downregulated genes were observed to be associated with membrane docking, endosomal vesicular transport, and smoothened signaling pathway, and these also deregulate neuronal patterning (Figure 8b). In the case of the heart, upregulated genes were observed to be influencing immune response-regulating signaling pathway, phagocytosis, regulation of developmental growth, cellular carbohydrate metabolic processes, glycerophospholipid biosynthetic process, multicellular organism growth and regulatory processes, positive regulation of small molecule metabolic process, etc. (Figure 9a). Downregulated proteins in the heart were observed to influence positive regulation of intracellular protein transport, cholesterol biosynthetic process, endosome to lysosome transport, negative regulation of calcium ion transport, secondary alcohol biosynthetic process, selective autophagy, sterol biosynthetic process, etc. (Figure 9b). Genes upregulated in the lung were observed to influence biosynthetic processes of cholesterol, sterol, and secondary alcohol (Figure 10a). ERK1 and EFRK2 cascade and its negative regulation, macroautophagy, vacuolar transport, vesicle organization, nucleus organization, endosome organization, membrane docking, protein K48-linked ubiquitination, etc., were observed to be influenced by downregulated genes (Figure 10b) (Supporting Information Data 7 and 8).

### 3.4 ORF3a Can Influence Biological Processes of a Tissue through miRNAs

**Figure 11:**
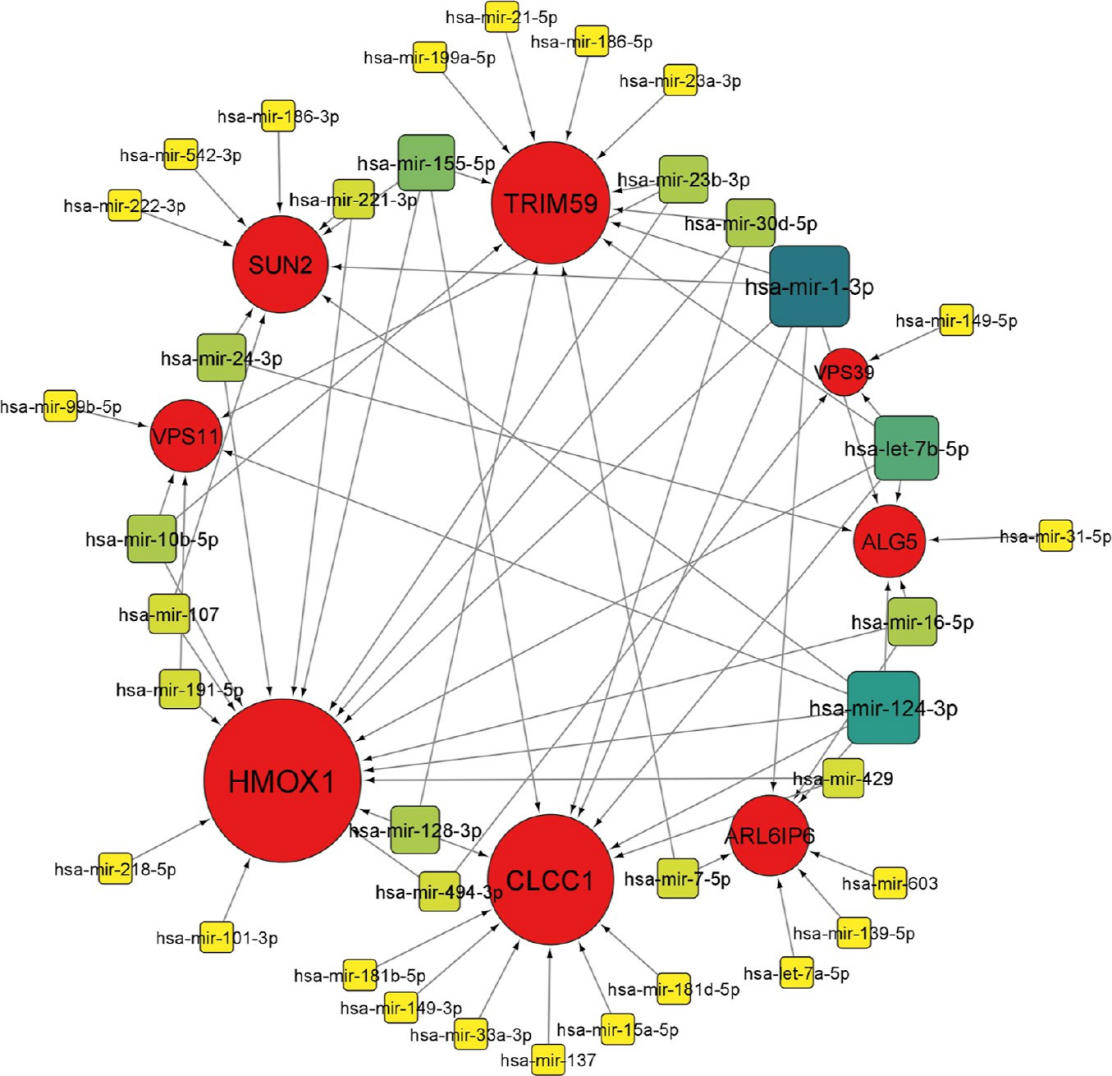
miRNA interaction network with SARS-CoV-2 ORF3a binding 8 proteins in the brain. Circles represent ORF3a binding proteins, and squares represent miRNAs. The increased size of nodes represents a higher degree. For miRNA, darker shades of continuous mapping of node color represent higher significance (dark green to yellow).

**Figure 12:**
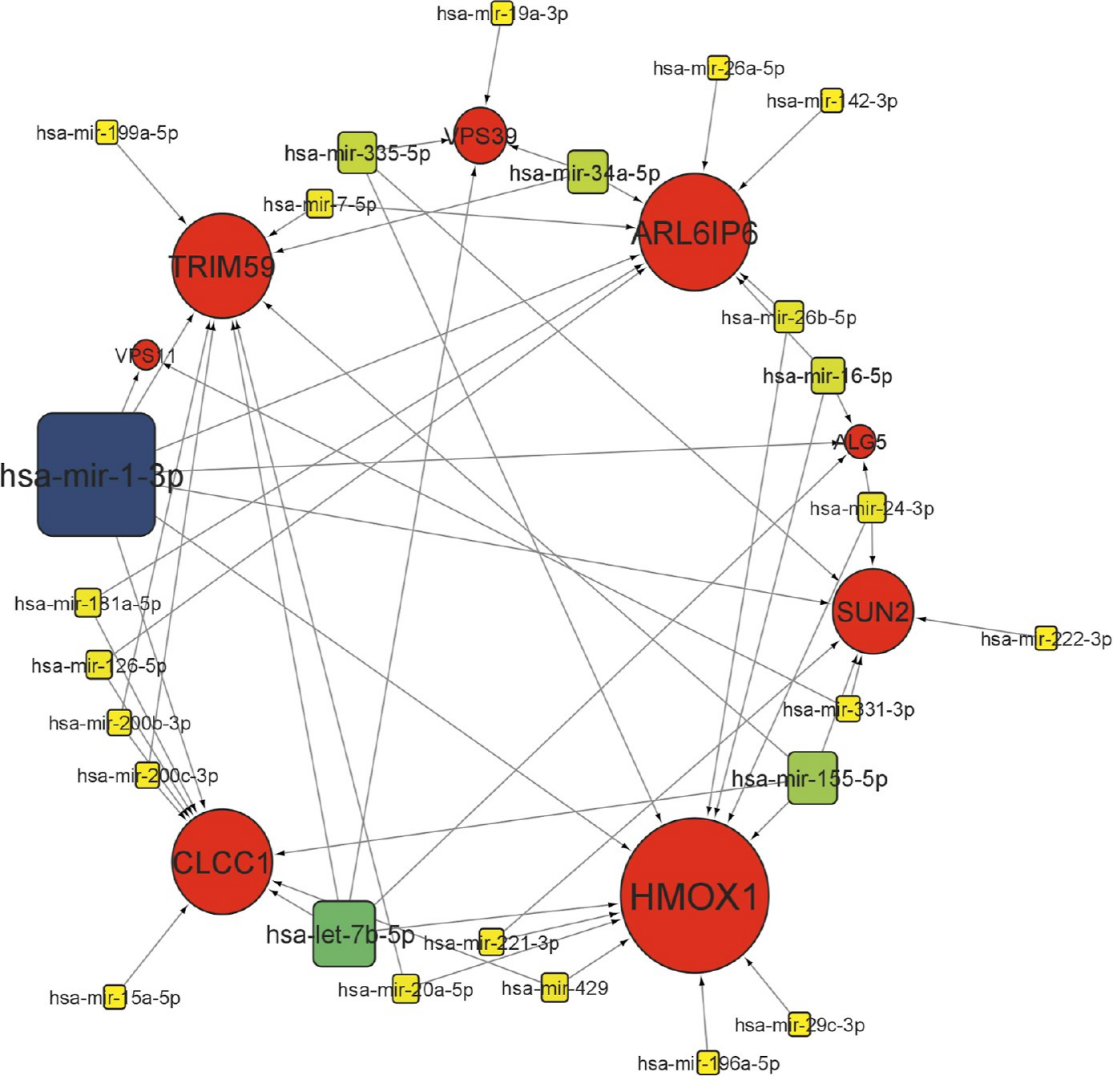
miRNA interaction network with SARS-CoV-2 ORF3a binding 8 proteins in the lung. Circles represent ORF3a binding proteins, and squares represent miRNAs. The increased size of nodes represents a higher degree. For miRNA, darker shades of continuous mapping of node color represent higher significance (dark green to yellow).

As miRNAs influence protein levels, we wanted to find out miRNAs targeting SARS-CoV-2 ORF3a binding 8 proteins. From miRNet analysis, we have identified 37 and 25 microRNAs interacting with ORF3a interacting eight proteins in the brain (Figure 11) and in the lung (Figure 12), respectively. In tissue-specific miRNA interaction networks, we have identified three miRNAs with high degree and betweenness values (hsa-mir-1-3p with degree 7, betweenness 182.07, hsa-mir-124-3p with degree 6 and betweenness 122.92 and hsa-let-7b-5p with degree 5, betweenness 109.07) in the brain. Similarly, in the lung-specific miRNA interaction network, we have identified only one miRNA with high degree and betweenness values (hsa-mir-1-3p with degree 7, betweenness 116.887). This indicates that these miRNAs act as hubs (for their high degree) and play a very important role in the interaction network (for their high betweenness). Apart from the regulatory effect on cytoskeletal rearrangement and protein localization, these miRNAs may influence diverse processes including NF-κB induced immune signaling cascades, cytokine biosynthesis, angiogenesis, cofactor catabolism, cell migration regulations, spindle fiber organization, DNA damage responses and endothelial cell proliferation as well as influence transcription factors binding with DNA. In the brain, because of the greater number of miRNAs interacting with ORF3a interacting proteins, miRNA-mediated pathways are more diverse. We observed three unique miRNA-mediated biological pathways in the brain. These were protein N-linked glycosylation, cytokine metabolic process, and negative regulation of sequence-specific DNA binding transcription factor activity (Supporting Information Data 9, 10, 11, 12, 13, and 14).

### 3.5 Tissue-Specific miRNA and Protein Interaction Network of SARS-CoV-2-Influenced miRNAs and Proteins

**Figure 13:**
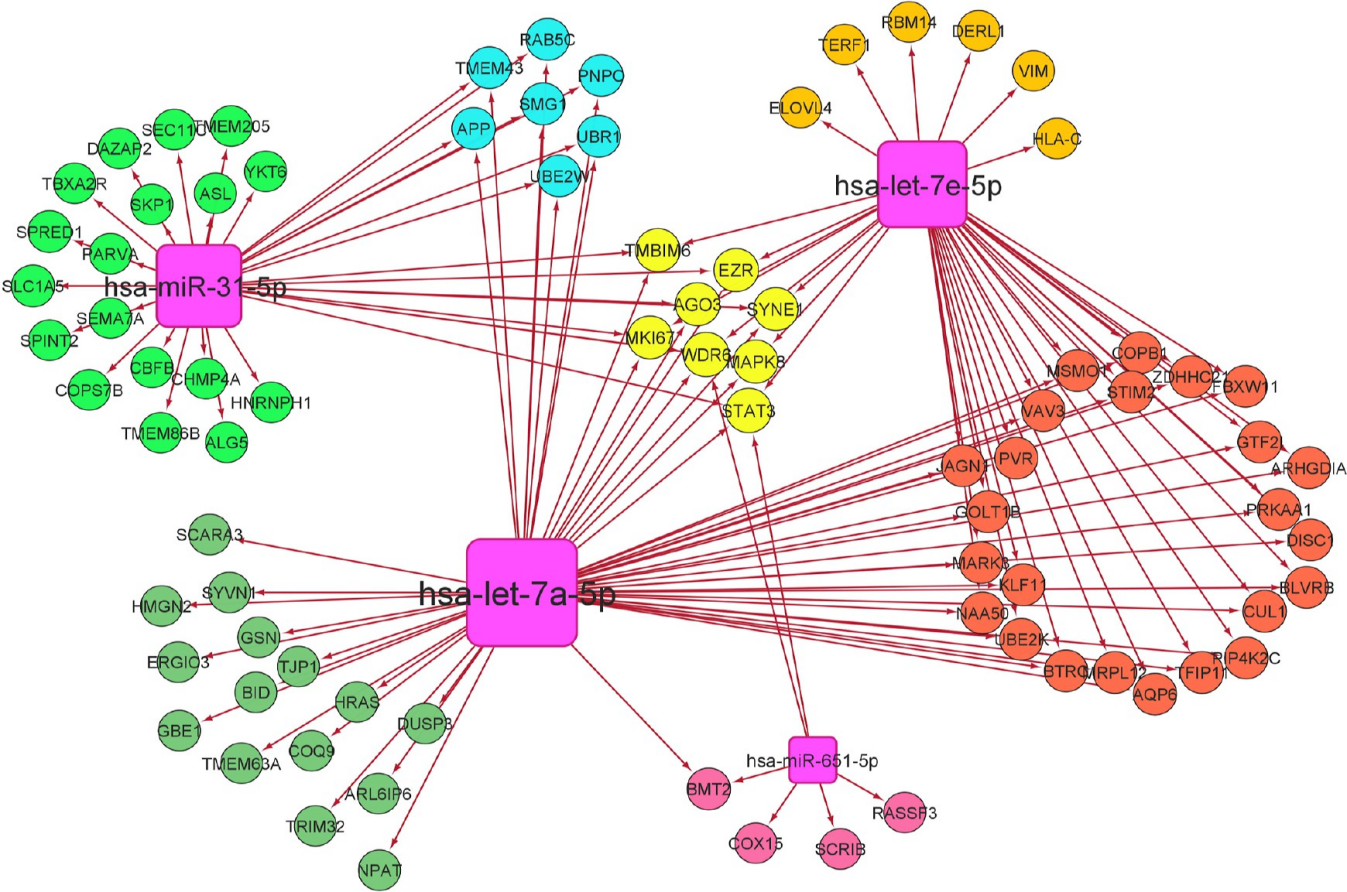
SARS-CoV-2-influenced miRNA—protein network in the brain. Common miRNAs in the reported list of SARS-CoV-2-influenced circulating miRNAs^60^ and the miRNAs that can target brain-expressing interacting partners of SARS-CoV-2 ORF3a interacting partners were taken for analysis. Proteins taken for analysis were SARS-CoV-2-influenced by SARS-CoV-2 ORF3a interacting proteins expressed in the brain. Nodes in circles are proteins, and nodes in squares are miRNAs.

**Figure 14:**
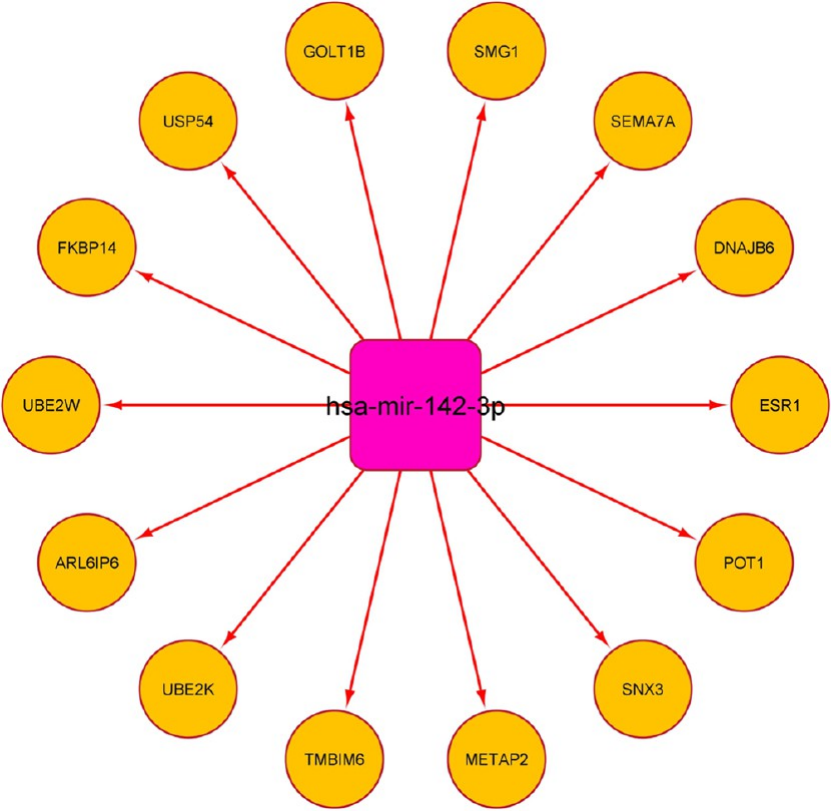
SARS-CoV-2-influenced miRNA—protein network in the lung. Common miRNAs in the reported list of SARS-CoV-2-influenced circulating miRNAs^60^ and the miRNAs that can target lung-expressing interacting partners of SARS-CoV-2 ORF3a interacting partners were taken for analysis. Proteins taken for analysis were SARS-CoV-2-influenced by SARS-CoV-2 ORF3a interacting proteins expressed in the lung. Nodes in circles are proteins, and nodes in squares are miRNAs.

To find out the effect of SARS-CoV-2 in host miRNA–ORF3a interacting protein network, we chose miRNAs that are reported to be regulated by SARS-CoV-2 with the following criteria: (i) miRNA should be present in the SARS-CoV-2-influenced circulating miRNA list,^60^ (ii) it should be expressed in our specific tissue of interest, and (iii) it should have a target in the expressed protein list of the tissue of interest that is influenced by SARS-CoV-2. To comply with the criteria, we first searched for miRNAs that can target the regulated proteins in miRNet for either the brain or lung. Only those miRNAs that were present in the SARS-CoV-2-influenced circulating miRNA list were taken for network analysis. SARS-CoV-2-influenced proteins that are interacting with SARS-CoV-2 ORF3a interacting 8 proteins expressed in either the brain or lung were taken for analysis to find out the miRNA-protein network in the respective tissues. In the brain, we found 4 miRNAs and they were hsa-let-7a-5p, hsa-let-7e-5p, hsa-miR-31-5p, and hsa-miR-651-5p (written in the order of decreasing degree). Among the proteins, WD repeat-containing protein 6 (WDR6) and signal transducer and activator of transcription 3 (STAT3) were targeted by all 4 miRNAs (Figure 13). In the case of the lung, we could find only one miRNA hsa-mir-142-3p that was targeting many proteins (Figure 14). Common miRNAs that were observed to be targeting ORF3a interacting 8 proteins and interacting with proteins influenced by SARS-CoV-2 were hsa-let-7a-5p (targeting ARL6IP6) and hsa-mir-31-5p (targeting ALG5); in the brain and in the lung, it was hsa-mir-142-3p (targeting ARL6IP6). Among the top 5 biological processes that could be regulated in the brain are intracellular protein transport, tube morphogenesis, viral reproductive process, cellular macromolecule catabolic process, and regulation of cellular protein metabolic process (Supporting Information Data 15). In the lung, we could find only two biological processes—I-kappaB kinase/NF-kappaB cascade and regulation of I-kappaB kinase/NF-kappaB cascade—with high significance (*p*-value ≤0.01) (Supporting Information Data 16).

## 4 Conclusions and Discussion

Tissue-specific complications upon SARS-CoV-2 infection were delineated in several analyses. However, the individual influence of SARS-CoV-2 accessory proteins is yet to be delineated in detail. Here we analyzed human proteins interacting with ORF3a viroporin and extended the analyses by incorporating other interacting proteins interacting with ORF3a viroporin and miRNAs interacting with ORF3a interacting proteins to get a global insight into ORF3a-mediated responses in host cells. SARS-CoV-2 infection and expression of ORF3a in host cells can result in the initiation of general and tissue-specific biological processes. We could find NOTCH1 and HRAS as hub genes in the common protein networks of regulated proteins after SARS-CoV-2 infection in the brain, lung, and heart. NOTCH1 was also reported to be a major hub gene that plays multiple roles in SARS-CoV-2.^64,65^ HRAS was observed to be involved in the SARS-CoV-2 immune response by peripheral blood mononuclear cells.^66^ In our study, ORF3a interacting proteins were observed to be influencing pathways involved in the regulation of autophagy, macroautophagy, and regulation of macroautophagy in the brain, heart, and lung. It is reported that patients with severe COVID-19 had significant impairment in antigen presentation with reduced expression of autophagy markers.^67,68^ Autophagy is one of the processes reported to be involved in MHC class I and II peptide presentation, thereby influencing antigen presentation.^69^ Induction of incomplete autophagy by ORF3a was shown to be through unfolded protein response.^70^ ORF3a and ORF7a were reported to be colocalizing in late endosomes and preventing their acidification.^71^ Strong protein-specific immunostaining of ORF3a was reported in the plasma membrane and endosomes of SARS-CoV-2-infected Caco-2 cells.^72^ ORF3a was observed to prevent the fusion between lysosomes and autophagosomes, whereas ORF7a reduces autophagosomal degradation by reducing the lysosome acidity.^71^ ORF3a inhibiting the fusion of autophagosomes with lysosomes is also reported.^49^ Other biological processes we observed to be influenced by OFR3a in the heart, lung, and brain are related to membrane and endomembrane systems. Biological processes involving vesicle organization, membrane docking, membrane fusion, vesicle fusion, organelle membrane fusion, and endosome organization came into this category. SARS-CoV-2 ORF3a was reported to be localized in both membrane and cytosolic fractions^46^ like immunostaining that was shown in these compartments.^72^ It is also observed to be localized on late endosomes and unlike SARS-CoV ORF3a, directly interacts with HOPS component VPS39 leading to the prevention of HOPS interaction with autophagosome-localized STX17 culminating in the formation of autolysosome.^48^ In a later study, it had been depicted that systemic inflammation results in the dysregulation of autophagy and neuroinflammation,^67^ as well as increased protein ubiquitination in SARS-CoV-2 infection.^73^ Our study adds the influence of ORF3a in K48-linked polyubiquitination as another common cellular event in SARS-CoV-2 infected in the brain, heart, and lung. A proteomic study comparing ubiquitin-modified proteome of SARS-CoV-2 infected cells speculates the modulation of K48-linked polyubiquitination to increase USP5 expression and type I IFN signal inhibition.^73^ In the brain, the effect of ORF3a can be severe as ORF3a interacting proteins influence cilia organization and viral budding. These effects can initiate other tissue-specific implications that increase susceptibility to other diseases.

Another observation that we had in specific biological processes in the heart was the negative regulation of calcium ion transport. Among COVID-19 patients treated with (298) and without (568) calcium channel blockers (CCBs), it was found that patients treated with CCBs had a significantly elevated rate of intubation.^74^ In another study among COVID-19 patients with a history of hypertension on dihydropyridine CCBs (70/245) and without CCBs (170/245), there was a significant increase in the risk for intubation or death among those who were taking dihydropyridine CCBs.^75^ Another study showed that chronic CCB users were more likely to be hospitalized with COVID-19 in comparison with long-term angiotensin-converting enzyme inhibitors- or angiotensin receptor blockers-using patients.^76^ Proteins involved in the biological process of negative regulation of calcium ion transport are voltage-dependent anion-selective channel protein 1 (VDAC1), transmembrane BAX inhibitor motif containing protein 6 (TMBIM6), and caveolin-3 (CAV3) where all three are downregulated. VDAC1 transports cations including Ca^2+^ ions in a low conductance state.^77^ TMBIM6 was observed to be downregulated in SARS-CoV-2-infected cells^78^ which may prompt Ca^2+^ disorder in the cells.^78^ CAV3 was shown to regulate ion channels in the caveolae of cardiac cells.^79^ L-type CCBs were shown to inhibit SARS-CoV-2 entry and infection in Vero E6 and Calu-3 cell cultures.^80^ Repurposing these drugs could have deleterious effects evidenced by observed reports^74−76^ and our observation also seems to be backing the observed reports. Though further in-depth studies are necessary to investigate the role of ORF3a in this aspect, the cumulative effect of downregulation of these three proteins could play an important role in the observed deleterious effect of CCBs in the context of SARS-CoV-2 infection. Our analyses included three data sets, and even with that we were able to find many tissue-specific biological processes. Detailed transcriptomics analyses of a large number of COVID-19 patients could unravel the tissue-specific influence of ORF3a in the severity of COVID-19 infection in much more detail.

## Supporting Information Available

Biological processes associated with clusters 1, 2, 3, 4, and 5 (PDF)
Data 1 All interacting proteins (XLSX)
Data 2 Hub genes (XLSX)
Data 3 PPI network with cluster parameters (XLSX)
Data 4 Various clusters (XLSX)
Data 5 ORF3a-related all DEGs’ biological pathways (XLSX)
Data 6 ORF3A-related common and unique pathways (XLSX)
Data 7 ORF3a DEGs’ biological pathways (XLSX)
Data 8 ORF3a related all DEGs in the heart, lungs, and brain (XLSX)
Data 9 Brain mirnet_enrichment (XLSX)
Data 10 Brain mirnet_mir_gene (XLSX)
Data 11 Brain node_table_mirnet (XLSX)
Data 12 Lung mirnet_enrichment (XLSX)
Data 13 Lung mirnet_mir_gene (XLSX)
Data 14 Lung node_table_mirnet (XLSX)
Data 15 Brain_mirnet_enrichment_ORF3a int proteins (XLSX)
Data 16 Lung_mirnet_enrichment_ORF3a int proteins (XLSX)
