# Supplementary material for: COVID-19 ORF3a Viroporin-Influenced Common and Unique Cellular Signaling Cascades in Lung, Heart, and the Brain Choroid Plexus Organoids with Additional Enriched MicroRNA Network Analyses for Lung and the Brain Tissues
Source: ACS Omega. 2023 Nov 17;8(48):45817–33. doi: 10.1021/acsomega.3c06485 (PMC10701872; doi:10.1021/acsomega.3c06485)
Supplement: Supplementary file 1 — ao3c06485_si_001.pdf [file ao3c06485_si_001.pdf]

Supplementary Figures

COVID-19 ORF3A VIROPORIN INFLUENCED COMMON AND UNIQUE CELLULAR SIGNALLING CASCADES IN LUNG, HEART AND BRAIN CHOROID PLEXUS ORGANOIDS WITH ADDITIONAL ENRICHED MICRORNA NETWORK ANALYSES FOR LUNG AND BRAIN TISSUES

SOURA CHAKRABORTY[‡,#]

School of Biotechnology, Jawaharlal Nehru University, New Mehrauli Road, New Delhi, India – 110067

SHRABONTI CHATTERJEE[‡]

Integrated Science Education and Research Centre (ISERC), Institute of Science (Siksha Bhavana), Visva Bharati (A Central University), Santiniketan (PO), Birbhum (DT), West Bengal, India – 731235

SUBHASHREE MARDI[†]

Integrated Science Education and Research Centre (ISERC), Institute of Science (Siksha Bhavana), Visva Bharati (A Central University), Santiniketan (PO), Birbhum (DT), West Bengal, India – 731235

JOYDEEP MAHATA[†]

Integrated Science Education and Research Centre (ISERC), Institute of Science (Siksha Bhavana), Visva Bharati (A Central University), Santiniketan (PO), Birbhum (DT), West Bengal, India – 731235

SUNEEL KATERIYA

School of Biotechnology, Jawaharlal Nehru University, New Mehrauli Road, New Delhi, India – 110067

PRADEEP PUNNAKKAL

Department of Biophysics, Postgraduate Institute of Medical Education & Research (PGIMER), Chandigarh, India – 160012

GIREESH ANIRUDHAN*

Integrated Science Education and Research Centre (ISERC), Institute of Science (Siksha Bhavana), Visva Bharati (A Central University), Santiniketan (PO), Birbhum (DT), West Bengal, India – 731235
gireesh1@gmail.com

Correspondence: gireesh1@gmail.com

‡ Authors contributed equally to the work
† Authors contributed equally to the work
# Current address: Division of Immunology, Department of Pathology, University of Cambridge, Cambridge, CB2 1QP, UK.

*Corresponding author and Corresponding author ORCID iD: https://orcid.org/0000-0001-7093-0404

List of Contents

Sr. No.	Description	Page No
1	**Figure S1.** Cluster 1 (Score – 7.707 with 42 nodes and 158 edges). MKI67 and EZR are two seed proteins. This complex is responsible for regulating cellular processes like cell cycle, cell death, apoptosis and cellular metabolic process. Yellow coloured box denotes seed protein. Continuous mapping of node colour signifies maximum score with darkest shade (dark pink) to least significant with lightest shade.	S3
2	**Figure S2.** Cluster 2 (Score – 6.182 with 56 nodes and 170 edges). UVRAG and EZR are two important seed proteins in this cluster that are involved in cellular localization, protein localization, transport process as vesicle mediated transport and protein transport. Yellow coloured box denotes seed protein. Continuous mapping of node colour signifies maximum score with darkest shade (dark green) to least significant with lightest shade.	S4
3	**Figure S3.** Cluster 3 (Score – 3.600 with 21 nodes and 36 edges). TCTN2 is acting as seed protein in this cluster regulating protein transport and localization processes. Yellow coloured box denotes seed protein. Continuous mapping of node colour signifies maximum score with darkest shade (dark orange) to least significant with lightest shade.	S5
4	**Figure S4.** Cluster 4 (Score – 3.600 with 6 nodes and 9 edges). MSMO1 is the seed protein in this cluster regulating lipid and steroid biosynthesis along with alcohol, sterol, lipid and steroid metabolic process. Yellow coloured box denotes seed protein. Continuous mapping of node colour signifies maximum score with darkest shade (dark green) to least significant with lightest shade.	S6
5	**Figure S5.** Cluster 5 (Score - 3.481 with 28 nodes and 47 edges). Seed protein of this cluster is EZR. Biological process annotation reveals cellular signalling and transduction and organ development processes. Yellow coloured box denotes seed protein. Continuous mapping of node colour signifies maximum score with darkest shade (dark violet) to least significant with lightest shade.	S7

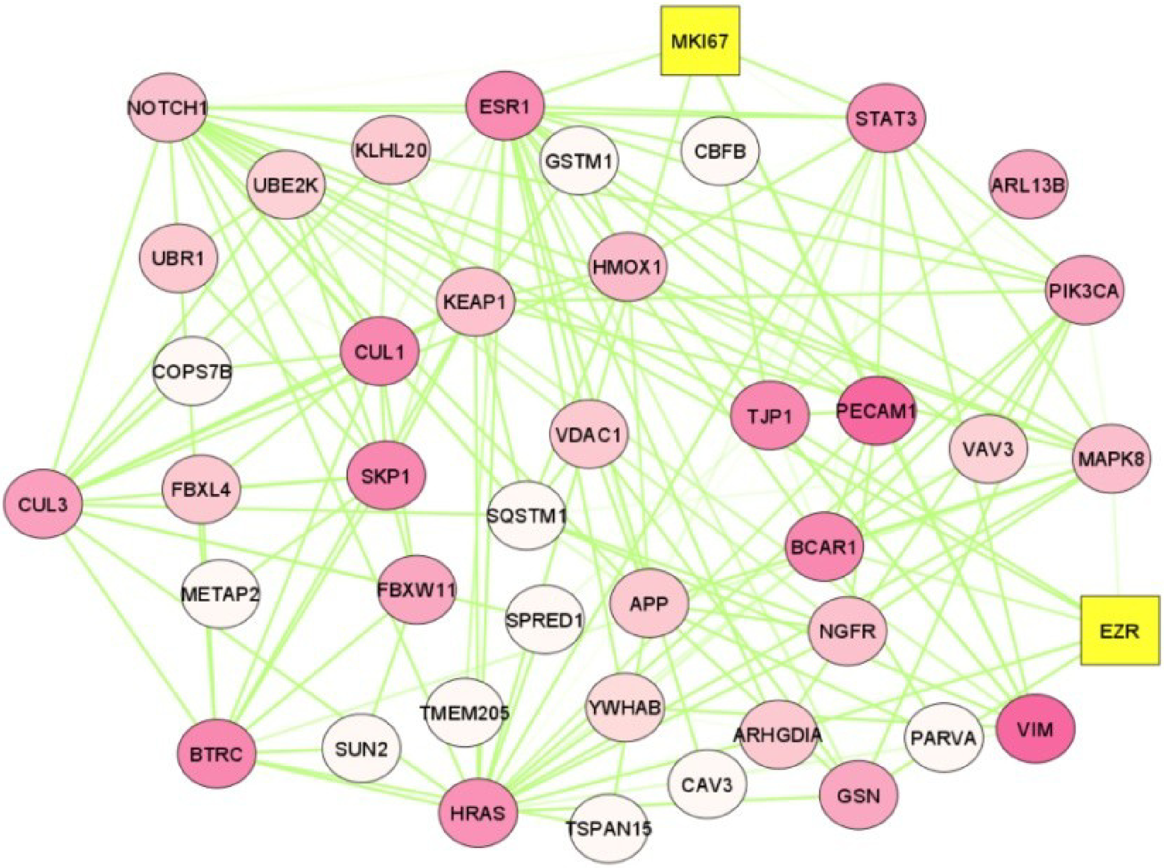

Figure S1. Cluster 1 (Score – 7.707 with 42 nodes and 158 edges). MKI67 and EZR are two seed proteins. This complex is responsible for regulating cellular processes like cell cycle, cell death, apoptosis and cellular metabolic process. Yellow coloured box denotes seed protein. Continuous mapping of node colour signifies maximum score with darkest shade (dark pink) to least significant with lightest shade.

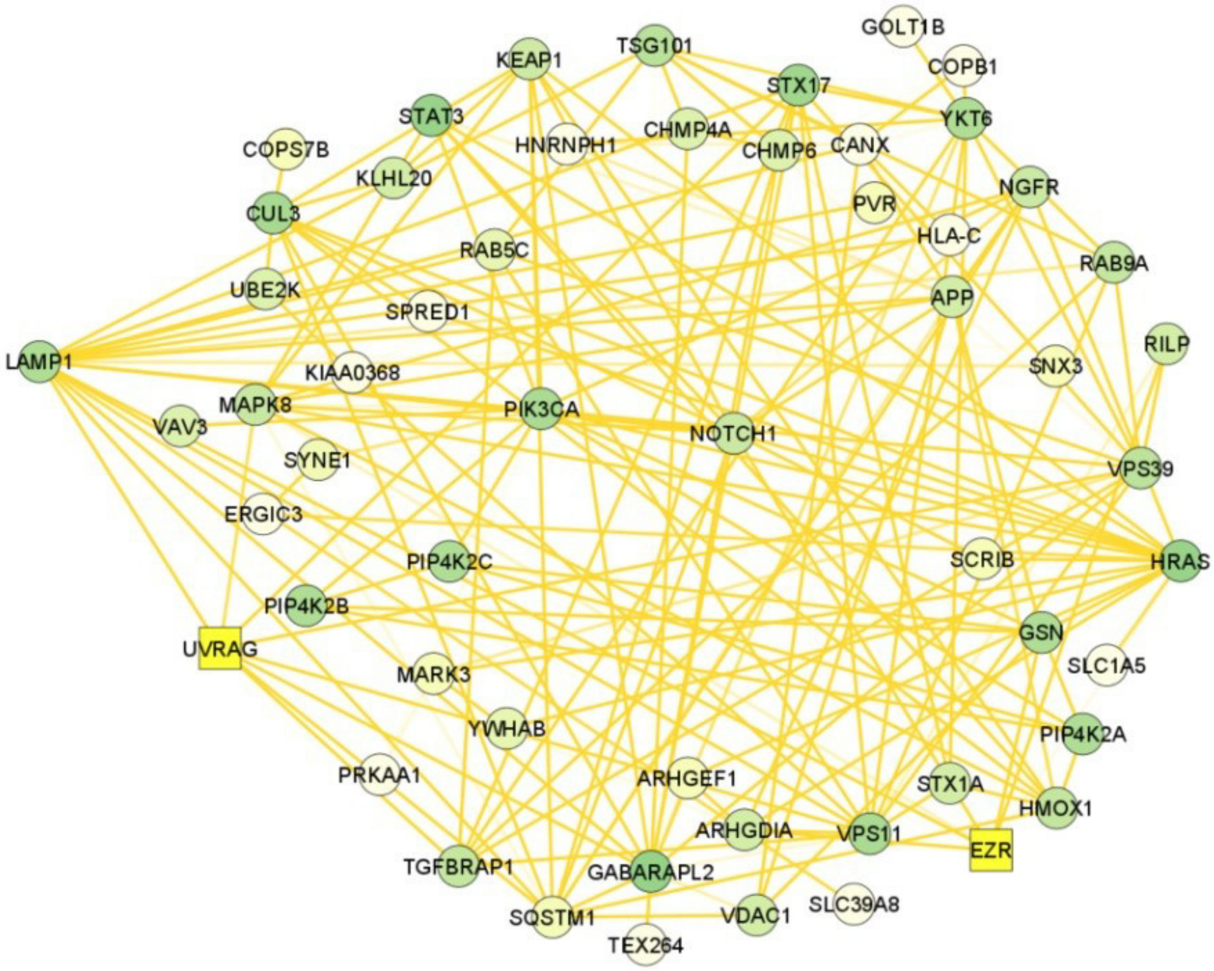

Figure S2. Cluster 2 (Score – 6.182 with 56 nodes and 170 edges). UVRAG and EZR are two important seed proteins in this cluster that are involved in cellular localization, protein localization, transport process as vesicle mediated transport and protein transport. Yellow coloured box denotes seed protein. Continuous mapping of node colour signifies maximum score with darkest shade (dark green) to least significant with lightest shade.

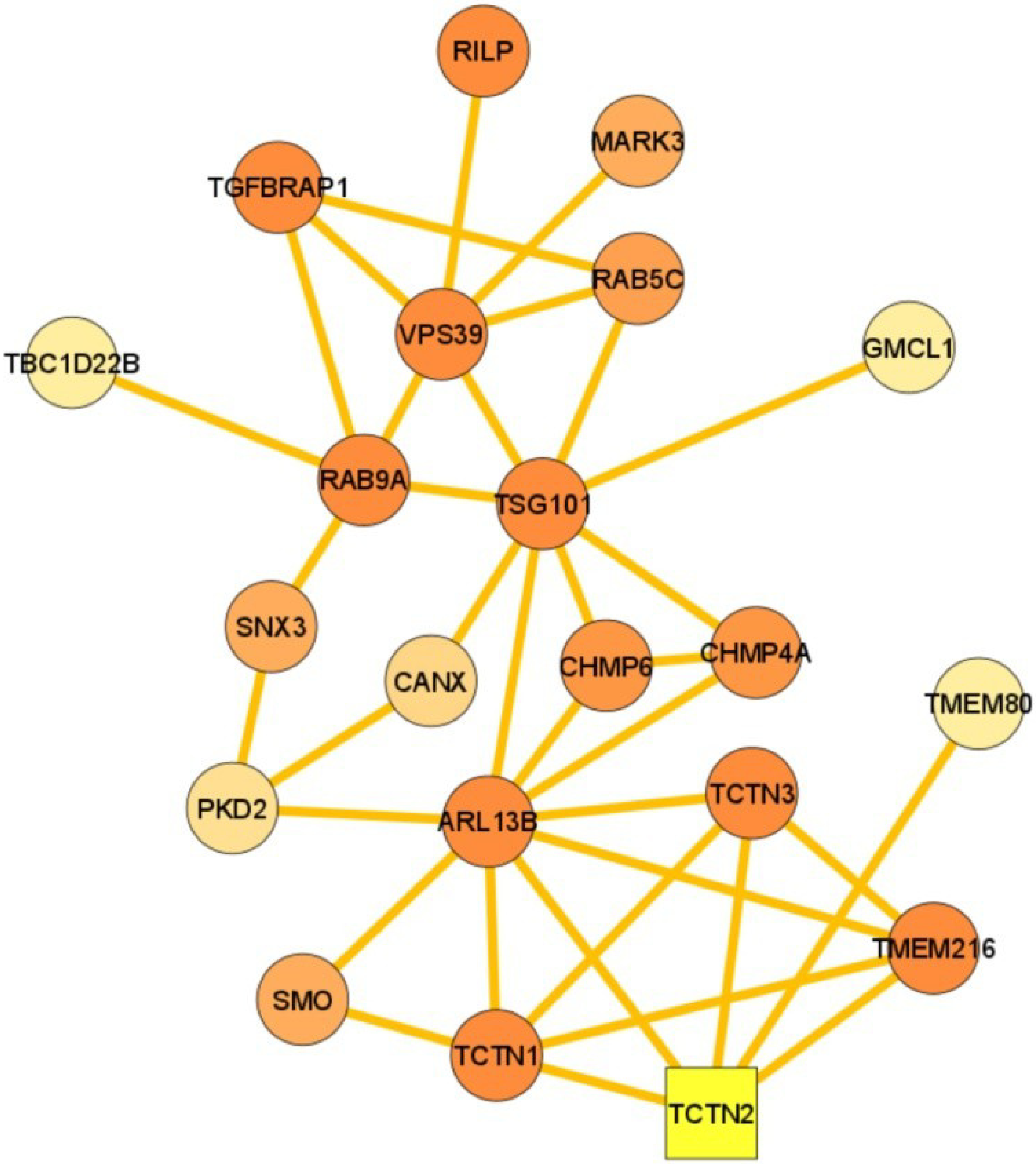

Figure S3. Cluster 3 (Score – 3.600 with 21 nodes and 36 edges). TCTN2 is acting as seed protein in this cluster regulating protein transport and localization processes. Yellow coloured box denotes seed protein. Continuous mapping of node colour signifies maximum score with darkest shade (dark orange) to least significant with lightest shade.

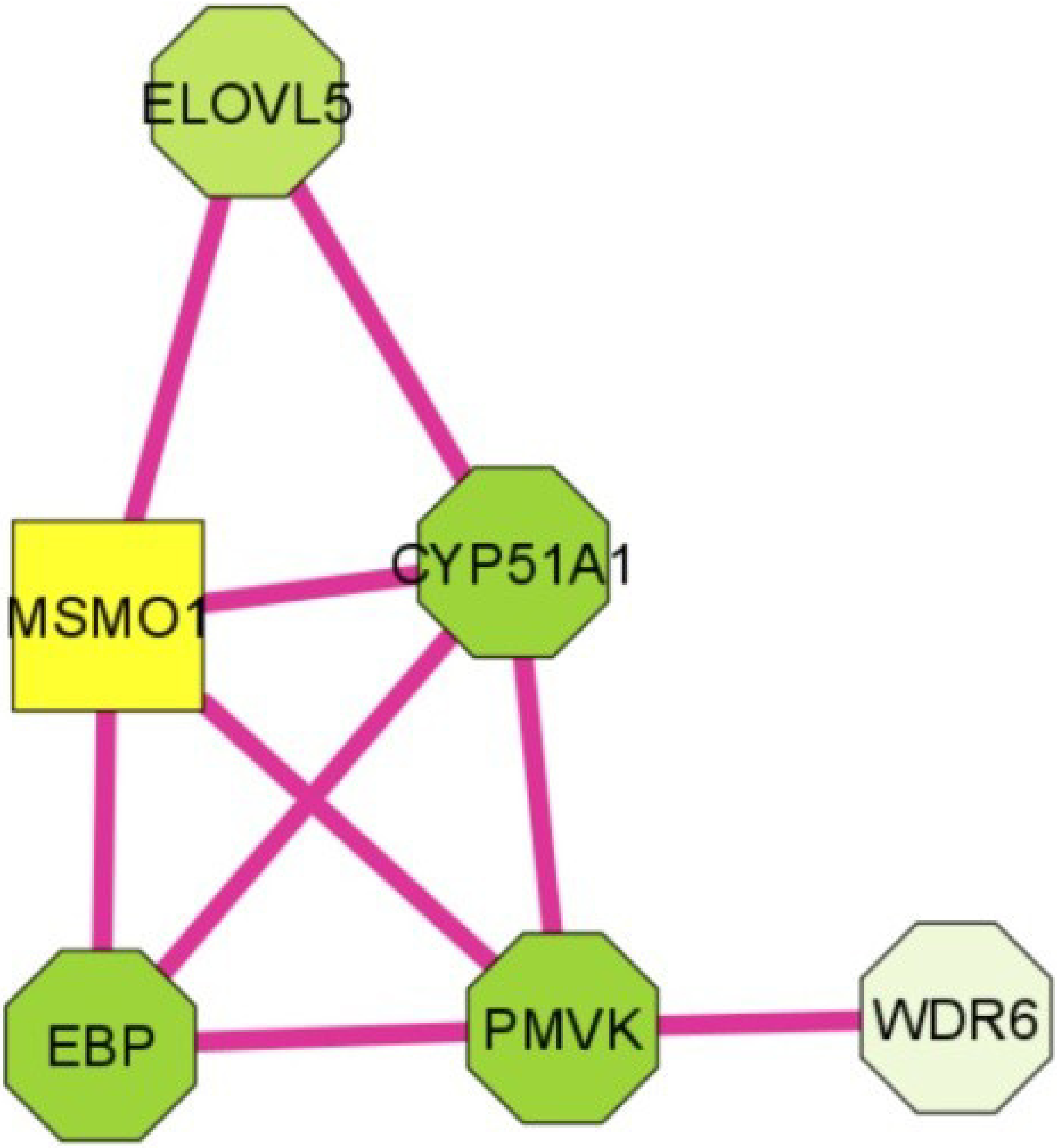

Figure S4. Cluster 4 (Score – 3.600 with 6 nodes and 9 edges). MSMO1 is the seed protein in this cluster regulating lipid and steroid biosynthesis along with alcohol, sterol, lipid and steroid metabolic process. Yellow coloured box denotes seed protein. Continuous mapping of node colour signifies maximum score with darkest shade (dark green) to least significant with lightest shade.

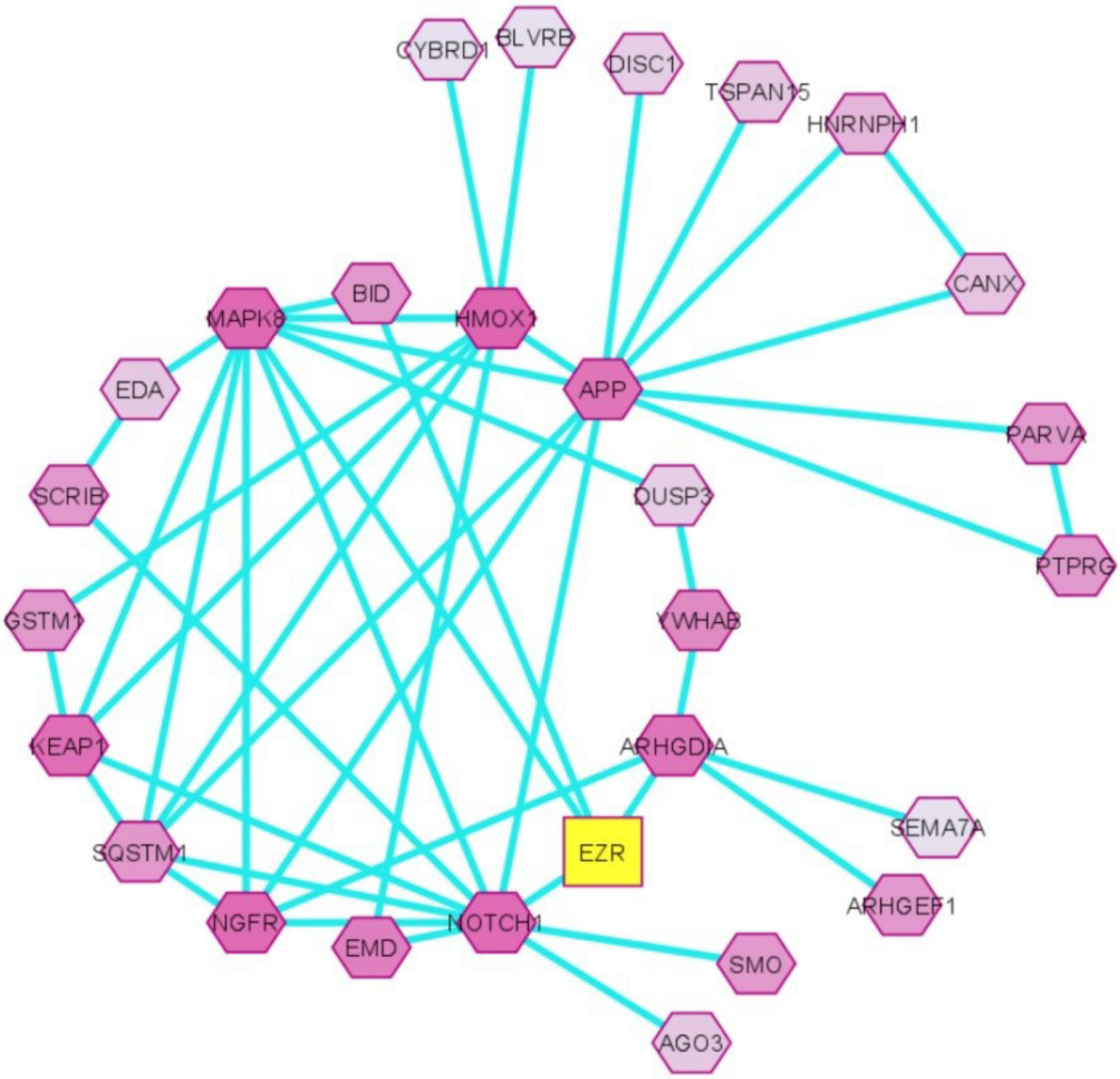

Figure S5. Cluster 5 (Score - 3.481 with 28 nodes and 47 edges). Seed protein of this cluster is EZR. Biological process annotation reveals cellular signalling and transduction and organ development processes. Yellow coloured box denotes seed protein. Continuous mapping of node colour signifies maximum score with darkest shade (dark violet) to least significant with lightest shade.
